# Development and psychometric evaluation of a tool to measure pain-management competency of surgical nurses

**DOI:** 10.3389/fmed.2026.1752781

**Published:** 2026-03-19

**Authors:** Yunxia Li, Zihao Xue, Shina Qiao, Yujun Lin, Xiaowen Fan, Meng Yang, Lihua Sun, Hongying Pan

**Affiliations:** 1 Nursing Department, Sir Run Run Shaw Hospital, Zhejiang University School of Medicine, Hangzhou, Zhejiang, China; 2 School of Public Health and Nursing, Hangzhou Normal University, Hangzhou, China; 3 Department of Anorectal Surgery, Affiliated Hospital of Youjiang Medical University for Nationalities, Baise, China; 4 Department of Pain Medicine, The Affiliated Hospital of Guizhou Medical University, Guiyang, Guizhou, China

**Keywords:** competency assessment, instrument development, pain management, psychometric validation, surgical nurses

## Abstract

**Introduction:**

Postoperative pain is a common symptom in surgical patients. Effective postoperative pain management can facilitate quick recovery of patients and enhance their comfort. Nurses' pain-management competency is crucial for ensuring the effective implementation of postoperative pain management; however, there is no effective tool to evaluate competency. In this study, we developed and validated the Surgical Nurses' Pain-Management Competency (SNPMC) tool for assessing surgical nurses' competency in pain management and evaluated competency levels and associated factors among Chinese surgical nurses.

**Methods:**

This study comprised two phases. (a) Measurement tool development, where items were generated through literature review and interviews, refined by expert discussions and two rounds of Delphi consultations, and finalized using the analytic hierarchy process. (b) Measurement validation and application, which evaluated content validity, construct validity, internal consistency, and test–retest reliability of the SNPMC. Using a multistage, geographically stratified convenience sampling strategy, 1,885 surgical nurses were surveyed from 48 hospitals across 15 regions representing the eastern, central, and western economic regions of China.

**Results:**

The final instrument included 78 items across seven dimensions: routine pain assessment, assessment and management of movement-evoked and unexpected pain, pharmacological pain management, patient-controlled analgesia management, non-pharmacological pain management, pain education, and professional development. The final SNPMC demonstrated excellent psychometric properties. Internal consistency was high (corrected item-total correlations: 0.672–0.847). Exploratory factor analysis supported the seven-dimensional structure (loadings ≥0.40). Content validity was strong (item-level content validity index: 0.80–1.00; scale-level content validity index: 0.98). The test–retest reliability over 14 days indicated strong stability. Chinese surgical nurses exhibited moderate to high levels of pain-management competency. Factors associated with competency included economic region, age, professional title, department, years of experience, education level, prior pain-management training, and prior pain management continuing education.

**Conclusion:**

The SNPMC effectively evaluates surgical nurses' pain-management competencies, highlighting the current competency levels and its contributing factors.

## 1 Introduction

The International Association for the Study of Pain (IASP) defines pain as “an unpleasant sensory and emotional experience associated with, or resembling, that associated with actual or potential tissue damage” (1). Despite advances in management, acute postoperative pain remains prevalent among surgical patients. A United States survey of 300 adults who underwent surgery in the past 5 years revealed that 86% experienced postoperative pain, with 75% reporting moderate to extreme pain (2). Similarly, a survey of 2,293 surgical patients in Southwest China found that 90.8% experienced pain at rest and 97.1% had pain during movement after surgery. Poorly managed acute pain can cause adverse physiological and psychological complications (3). Indeed, many patients develop chronic pain that significantly impairs their quality of life for years after surgery (4). Hence, pain negatively impacts individuals, healthcare system, and society. Uncontrolled pain can prolong hospitalization, increase mortality and morbidity rates, lead to repeat hospitalizations, and elevate costs for patients and the government (5, 6).

Postoperative pain occurs within a short and dynamic clinical window and is closely linked to function and recovery. The Chinese Nursing Association group defines standard postoperative pain as acute nociceptive pain from the end of surgery until discharge (7) and recommends functional activity-oriented evaluation (7). Moreover, It highlights practice demands such as timely reassessment after analgesic administration and safe nursing management of patient-controlled analgesia (PCA), including shift-based checks and patient instruction for button use. Consistent with perioperative consensus guidance that pain assessment should facilitate function rather than treat numerical scores alone (8), these features underscore the need to focus specifically on surgical nurses when assessing postoperative pain-management competency.

Effective pain management is a moral imperative and professional responsibility of healthcare providers (9), making competency in this area essential. The World Health Organization defines competency as “the observable ability of a person to integrate knowledge, skills, values, and beliefs in their task performance. Competencies are durable, trainable, and, through the expression of behaviors, measurable” (10). Nursing competency is the complex integration of knowledge, including professional judgment, skills, values, and attitudes (11). Mastery of pain management is essential for nurses given their pivotal roles in assessment, intervention, and patient support. Therefore, nurses must possess clinical competencies to assess and manage pain regardless of the population group or setting (12).

Assessing nurses' competency in managing postoperative pain is vital for evaluating care quality, ensuring high-standard practices, promoting patient safety, and improving outcomes. Research on nurses' pain-management competencies is limited. The Nurses' Cancer Pain Management Competency Scale (NCPMCS) (13) assesses nurses' skills in managing cancer-related pain based on the core pain-management competencies identified by Fishman et al. (14). It comprises four factors: pain management context, pain assessment and measurement, pain management, and multidimensional nature of pain. However, postoperative pain care involves time-sensitive bedside reassessment, function-oriented evaluation, and device-based analgesia workflows (e.g., PCA), which are not explicitly operationalized in cancer-pain competency instruments (7, 13, 14). Thus, the NCPMCS is unsuitable for assessing postoperative pain-management competency.

Existing pain-management research has primarily focused on the competencies of nursing students and nurses, particularly on their knowledge of pain management and skills in pain assessment. Herr et al. (15) integrated 21 pain-management competencies into the nursing education curriculum to ensure nursing graduates acquire essential pain-management skills. Although this tool addresses basic pain-management competencies within pre-licensure nursing education, it lacks a comprehensive assessment, such as evaluating the ability to manage complex pain cases.

A pain-management knowledge and attitude questionnaire for Chinese nurses was designed by Chen et al. (16), drawing on the Pain Nursing Certification Exam Outline (17), IASP's Pain Nursing Course Syllabus (18), validated Knowledge and Attitudes Survey Regarding Pain (19), and relevant policy guidelines in China. Although the questionnaire is a validated tool for measuring nurses' pain knowledge and attitudes, it is not suitable for assessing nurses' overall pain competency. Li et al. (20) developed a validated instrument to evaluate nurses' practical pain assessment abilities based on a literature review and Delphi expert consensus, targeting evidence-based pain assessment practices among medical-surgical and outpatient nursing staff. Although it gauges the current state of clinical pain assessment practices, it is not intended to comprehensively measure nurses' pain competencies. Despite their limitations in evaluating comprehensive pain-management competency, they offer valuable references for further research in this area.

Currently, these existing instruments focus mainly on cancer-pain contexts (13), prelicensure curriculum competencies (15), pain knowledge/attitudes (16), or pain-assessment subskills (20), and are conceptually narrower than required for assessment of nurses' competency in postoperative pain management. In contrast, postoperative surgical nursing practice requires integrated, behavior-based competencies aligned with perioperative guidance and nursing standards (7, 8). Therefore, this study aimed to develop and validate the Surgical Nurses' Pain-Management Competency (SNPMC) tool to support competency assessment, targeted education and training, and quality improvement in postoperative pain care (7, 8).

To enhance its practical utility, the SNPMC incorporates distribution-based normative percentile bands to facilitate tiered interpretation of competency levels and identification of areas requiring improvement. Domain-level scoring may inform targeted remediation pathways and curriculum mapping, whereas future implementation could integrate SNPMC results into digital dashboards for longitudinal tracking and exploration of associations with relevant quality indicators (e.g., reassessment adherence and PCA safety practices). Given its potential relevance beyond China, further cross-cultural adaptation and validation are warranted.

## 2 Materials and methods

**Figure 1:**
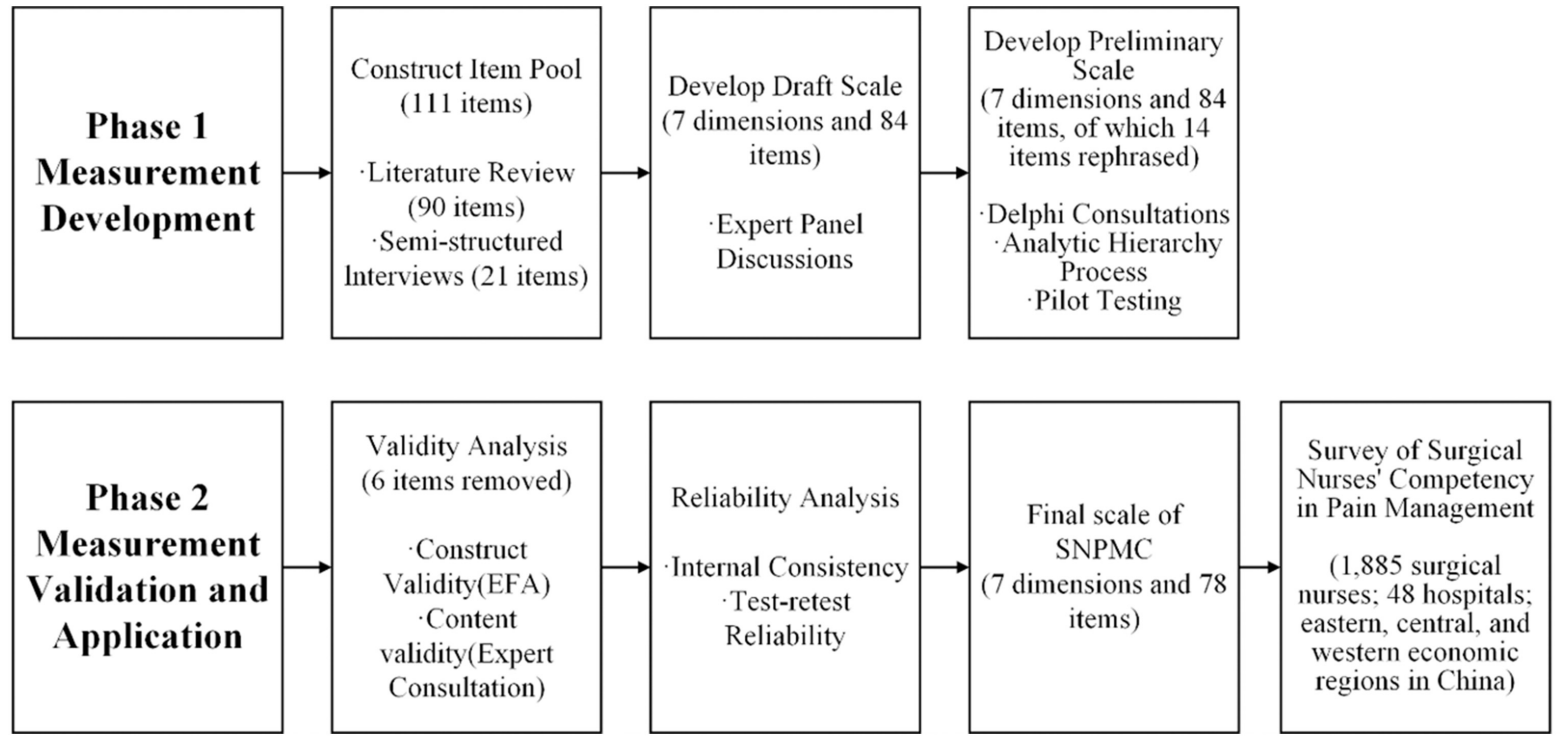
Research design flowchart.

The study was conducted in two phases (Figure 1). Phase 1 focused on measurement tool development, where initial items were generated through a literature review and interviews, refined via expert discussions and Delphi consultation, and finalized using the analytic hierarchy process (AHP). A pilot test was conducted to assess clarity and applicability. Phase 2 involved measurement validation and application via a cross-sectional survey. In this phase, a multistage, geographically stratified convenience sampling strategy was employed to recruit surgical nurses across China. Data were collected electronically, and statistical analyses including construct validity, internal consistency, and test–retest reliability assessments were performed. This study was approved by the Ethics Committee of Sir Run Run Shaw Hospital (Approval Number: 2024-0482).

### 2.1 Measurement development

#### 2.1.1 Item generation via literature review and qualitative interviews

A comprehensive literature review was conducted to identify potential items to include in the measurement instrument. Searches were conducted in English databases (PubMed, Web of Science, Embase, and CINAHL) and Chinese databases (CNKI and SinoMed). The keywords used in the search strategy were “postoperative pain,” “acute pain,” “chronic postoperative pain,” “pain,” “pain management,” “pain assessment,” “pain control,” “nursing,” “nursing practice,” “expert consensus,” “guidelines,” “nurse,” “surgical nurse,” “nurse role,” “competency,” and “core competence.” The literature search was limited to the period between January 2000 and December 2023. The inclusion criteria for studies were as follows: (a) addressed pain-management nursing as the central theme; (b) included the scope of pain management nursing work performed by surgical nurses; (c) investigated postoperative pain-management protocols; and (d) cultivated nurses' pain-management capabilities. Exclusion criteria comprised: (a) unavailable full text; (b) non-English or non-Chinese publication language; (c) conference paper, book, or research proposal. A total of 8,636 articles were retrieved. After removal of duplicate entries, titles/abstracts were screened against the predefined eligibility criteria, and potentially relevant records underwent full-text assessment. The overall development workflow is summarized in Figure 1; the literature review contributed 90 candidate items from 34 included articles.

Semi-structured interviews were conducted to gather information incompletely captured in the literature review. The 12 interview participants were surgical nurses with at least 5 years of clinical experience in surgical nursing. The main interview questions were: (a) In your view, what key qualities or competencies should surgical nurses possess when providing pain management?; and (b) Based on your experience, what core domains should be considered when evaluating the performance of surgical nurses in pain management? Data saturation was achieved when no new themes or significant information emerged from the interviews. The 12 participants represented diverse clinical departments, including orthopedics (*n* = 4, 33.3%), urology (*n* = 3, 25.0%), general surgery (*n* = 2, 16.7%), anorectal surgery (*n* = 1, 8.3%), pain clinics (*n* = 1, 8.3%), and intensive care unit (*n* = 1, 8.3%). Their work experience ranged from 9 to 33 years, with a mean of 17.6 ± 7.6 years. Using content analysis, 21 items were extracted from the interview data. Therefore, 111 items were extracted after the literature review and semi-structured interviews.

#### 2.1.2 Item refinement using expert panel discussion and the Delphi method

Two group expert panel discussion sessions, each lasting approximately 1 h, were conducted. The discussion group comprised five nurses aged 28–49 years. Their professional backgrounds included advanced practice nursing specializing in pain management (*n* = 2), orthopedic nursing (*n* = 1), post-anesthesia care (*n* = 1), and nursing education (*n* = 1). These sessions discussed the initial item pool generated from the literature review (*n* = 90) and semi-structured interviews (*n* = 21). The items were classified, merged, and deleted, generating the preliminary draft of the evaluation tool comprising seven dimensions and 84 items (Table 1).

**Table 1:** Dimensions of surgical nurses' pain management competence.

**Dimension**	**Meaning**
Dimension A: routine pain assessment	Essential practices of systematically assessing patients' pain: timing and frequency of assessments, content and characteristics evaluated, use of appropriate assessment tools, individualized assessment approaches, continuous monitoring, and accurate documentation of pain findings.
Dimension B: assessment and management of movement-evoked pain and unexpected pain	Critical assessment and timely management of specific pain types that require prompt attention: pain provoked by movement or activity (movement-evoked pain), pain that is sudden, severe, worsening, or otherwise unexpected (breakthrough or abnormal pain). Identifies underlying causes requiring immediate interventions or reporting to the medical team.
Dimension C: pharmacological pain management	Nursing responsibilities related to pharmacological pain management: administration of prescribed analgesics per orders, monitoring analgesic effectiveness and potential side effects, preventing common adverse events associated with pain medications, and managing/reporting drug-related issues or complications in the context of systemic and regional analgesia.
Dimension D: patient-controlled analgesia (PCA) management	Specific nursing care associated with PCA management: checking and maintaining PCA equipment, instructing patients on proper use, monitoring patients' responses, assessing effectiveness and potential complications or side effects specific to PCA, and troubleshooting pump issues.
Dimension E: non-pharmacological pain management	Application of non-pharmacological interventions for pain relief and comfort: implementing strategies such as position changes, providing a comfortable environment, using physical methods, and facilitating psychological and behavioral approaches.
Dimension F: pain education	Crucial role of nurses in educating patients and their families about topics related to pain and its management: pain assessment; pain medication use, potential side effects, and safe disposal; application of non-pharmacological techniques; importance of functional exercise as it relates to pain; planning for pain management after discharge.
Dimension G: professional development competencies	Reflects the broader, higher-level professional competencies required for effective pain management beyond direct task execution: collaborating within multidisciplinary teams; utilizing evidence-based practices; continuous learning and innovation in clinical knowledge, engaging in quality improvement initiatives based on assessment outcomes; understanding the physiological mechanisms of pain; developing and adjusting complex, individualized care plans based on patient factors and assessment results.

The Delphi expert consultation method was employed to refine the items and determine their weights. The inclusion criteria for the experts in the consultation were as follows: (a) engaged in specialized nursing and nursing management work for 8 or more years, (b) hold a bachelor's degree or higher, and (c) hold an intermediate or higher professional title.

The expert consultation questionnaire contained three sections: (a) general demographic information; (b) expert consultation form using a five-point Likert scale to evaluate indicator importance (1 = unimportant to 5 = very important); and (c) expert familiarity and judgement basis form, including revision suggestions following each indicator level.

In the Delphi process, the participants in Round 1 rated the importance of the SNPMC preliminary draft. After receiving expert responses, opinions were organized and summarized, and the questionnaire was revised. Inclusion criteria for items were a mean score ≥4 and coefficient of variation (CV) ≤ 0.25. Based on expert input, items were added, merged, or revised. Round 2 was conducted 4 weeks later based on feedback from Round 1. In this round, the importance of each indicator was reevaluated. The Delphi survey concluded after two rounds upon opinion convergence.

#### 2.1.3 Finalization and weight assignment using expert panel review and AHP

Following the final round of Delphi consultation and determination of the core competency items, an expert panel convened to review and refine the items. Through expert panel discussions, the final competency items were converted to questionnaire format for data collection. The panel reviewed and refined the questionnaire items for clarity, readability, and appropriateness before deployment.

The AHP was applied to determine the weight coefficients of each item, culminating in the final SNPMC. Using the Saaty scale, experts evaluated the relative importance of each pairwise combination of items to derive weight coefficients. The consistency of the pairwise judgments was confirmed at each level of the hierarchy. All consistency ratios (CR) were found to be between 0 and 0.0463, well below the acceptable threshold of 0.10, indicating a high degree of reliability and logical consistency in the expert judgments.

#### 2.1.4 Pilot testing

Pilot testing was performed face-to-face with a convenience sample of surgical nurses to evaluate the understandability and applicability of the SNPMC. Participants were registered nurses with at least 1 year of service and a formal employment status at the surveyed hospital. Nurses were excluded if they were interns, visiting staff, or on leave (e.g., sick or parenting leave) during the survey period. Nurses completed the preliminary draft of the SNPMC and provided feedback on its applicability, focusing on factors such as completion time, overall length, item clarity and relevance, and instruction comprehensibility.

### 2.2 Measurement validation and application

#### 2.2.1 Psychometric testing

##### 2.2.1.1 Sampling design and participant recruitment

The required sample size was determined based on a preliminary version of the SNPMC, which comprised 84 items. Following the generally accepted guidelines of including at least 10 participants per item to ensure adequate statistical power for psychometric analysis (21), and accounting for an anticipated 20% invalid questionnaire rate, a minimum sample size of 1,020 surgical nurses was applied.

A multistage convenience sampling strategy was used to ensure representation across China's three major geographic and economic regions: eastern, central, and western (22). The eastern economic region is generally more developed and includes coastal provinces, encompassing inland provinces with developing industries. In contrast, the western economic region comprises provinces with vast land areas and relatively less developed economies. Although probability sampling is ideal, convenience sampling was selected because it was the most feasible and cost-effective method for reaching several surgical nurses across a wide geographic area.

The sampling process involved three steps. (a) Province selection: at least four provinces or autonomous regions were selected from each economic region. (b) Hospital selection: researchers contacted nursing directors or designated coordinators at potential hospitals in the selected regions. Upon agreement to participate, 3–8 public hospitals (Tier-2 or Tier-3) with a minimum of 500 beds were recruited per selected province or region. Primary care institutions (Tier-1 hospitals) were excluded. (c) Nurse recruitment: designated coordinators at each hospital distributed the electronic survey link to eligible surgical nurses via internal communication channels (e.g., WeChat work groups). Approximately 50 eligible surgical nurses were recruited from each hospital.

The inclusion criteria for recruited nurses were: (a) registered nurse with ≥1 year of clinical service in the surveyed hospital; (b) official hospital employee; and (c) working in a surgical ward during the survey period. Nurses were excluded if they were interns, visiting staff, or on leave during the data collection period. Following this sampling procedure, an estimated 720 to 1,760 surgical nurses were expected to participate, with a 10% response rate.

To assess the test–retest reliability, 28 surgical nurses from one hospital were selected using the same inclusion criteria. The SNPMC was administered twice to the participants, with a 14-day interval between measurements.

##### 2.2.1.2 Instruments

Data were collected using two instruments. The first was a general information questionnaire, collecting demographic information (sex, age, education, professional title, and position), work-related characteristics (department, years of service), and pain nursing training and policy-related factors (receipt of pain nursing training in the past year; availability of pain nursing policies or guidelines in the department). The second was the SNPMC, using the preliminary scale developed in phase 1. This instrument comprised items rated on a five-point Likert scale (1 = not competent at all, to 5 = completely competent), with higher scores reflecting greater pain-management competency.

#### 2.2.2 Data collection procedure

Each participating hospital appointed a trained investigator to administer the survey and obtain informed consent. Data were collected electronically via the Questionnaire Star platform (Chinese equivalent of Amazon Mechanical Turk), facilitating large-scale structured data collection. The questionnaires included a consent page, a general information section, and the SNPMC. Participants completed the online survey after providing informed consent.

### 2.3 Statistical analysis

Data were analyzed using SPSS version 26.0 (IBM Corp., Armonk, NY, USA), with the significance level at *p* < 0.05. Categorical variables are described using frequencies and percentages; continuous variables are described as means and standard deviations ($\bar{\text{x}}$ ± s). The normality of data distribution was assessed, and independent sample *t*-tests were used for normally distributed data, whereas Mann–Whitney *U*-tests were used for non-normally distributed data.

#### 2.3.1 Construct validity

Given that the SNPMC is a newly developed instrument, exploratory factor analysis (EFA) was employed to explore the underlying latent structure of the items and refine the dimensions, rather than to confirm a pre-existing theoretical model. EFA was separately conducted for each of the seven predefined SNPMC dimensions using varimax rotation. Prior to analysis, normality was assessed using P–P plot; the data were normally distributed, allowing EFA. The Kaiser–Meyer–Olkin (KMO) coefficient and Bartlett's test of sphericity were used to determine the factorability of the data for factor analysis. Items with factor loadings ≥0.40 were retained.

#### 2.3.2 Content validity

Content validity was evaluated using the content validity index (CVI), including the item-level CVI (I-CVI) and scale-level CVI (S-CVI), based on expert opinions. To calculate the I-CVI, panel members were asked to rate each item's relevance to the underlying construct on a four-point Likert-type scale (1 = not relevant, 2 = somewhat relevant, 3 = quite relevant, and 4 = highly relevant). Ratings of 1 and 2 indicated invalid content, whereas ratings of 3 and 4 indicated valid content (23). For each item, the I-CVI was calculated as the number of experts rating 3 or 4 divided by the total number of experts. To calculate the S-CVI, the average proportion of items rated as relevant across experts was calculated (23). I-CVI > 0.78 and S-CVI > 0.90 were considered acceptable criteria (24).

#### 2.3.3 Reliability testing

Reliability was assessed using internal consistency, item-total correlation, and test–retest stability. Cronbach's alpha and McDonald's omega were computed to assess internal consistency. A minimum Cronbach's alpha of 0.70 was considered satisfactory (25). Pearson's correlation coefficient was used to assess corrected item-total consistency; a threshold of *r* > 0.40 was set (26). Test–retest reliability was examined by comparing Pearson's correlation coefficients and intraclass correlation coefficient (ICC) between two time points (14-day intervals) among the 30 nurses.

## 3 Results

### 3.1 Delphi expert consultation and pilot testing results (phase 1)

Two rounds of consultation were conducted with 23 experts in each. The experts had a mean age of 46.0 ± 5.3 years and a mean working experience of 19.6 ± 9.1 years in nursing management, bedside nursing, nursing education, pain management nursing, and orthopedics, covering 13 provinces, municipalities, or autonomous regions in China. Table 2 summarizes the panel's demographic profile, illustrating their high level of seniority and diverse professional backgrounds.

**Table 2:** Characteristics of the Delphi expert panel (*n* = 23).

**Category**	**Count**
**Sex**, ***n*** **(%)**	
Male	1 (4.3)
Female	22 (95.7)
**Age (**$\bar{\text{x}}$ ±**s)**	46.0 ± 5.3
**Education level**, ***n*** **(%)**	
Bachelor's degree	16 (69.6)
Master's degree	4 (17.4)
Doctoral degree	3 (13)
**Professional title**, ***n*** **(%)**	
Intermediate	1 (4.3)
Associate senior	15 (65.2)
Senior	7 (30.4)
**Years of working experience (**$\bar{\text{x}}$ ±**s)**	19.6 ± 9.1
**Field of work (multiple choice)**, ***n*** **(%)**	
Nursing management	20 (87)
Bedside nursing	6 (26.1)
Nursing education	1 (4.3)
Pain management nursing	1 (4.3)
Orthopedics	1 (4.3)
**Economic region**, ***n*** **(%)**	
Eastern	15 (65.2%)
Central	7 (30.4%)
Western	1 (4.3%)

In the first round of expert consultation, the mean importance scores for all items ranged from 4.22 to 5.00, with CV values between 0 and 0.18. No item modifications were required. However, based on qualitative feedback from experts, revisions were made before the second round.

In the second round, the mean importance scores ranged from 4.35 to 5.00, with CVs from 0 to 0.23. The final framework comprised seven dimensions and 84 items. Table 3 outlines the finalized items and item weights. AHP was applied to the second-round data to determine the relative weight of each item, with weights ranging from 0.0011 to 0.0462.

**Table 3:** SNPMC item importance and weights.

**Dimension**	**Item**	**Mean of importance**	**SD**	**CV**	**Factor loading**	**Weight**	**Score**
Dimension A: routine pain assessment	• A-1 conduct pain assessment when a patient reports pain	4.96	0.21	0.04	0.706^b^	0.0117	1
	• A-2 conduct pain assessment before administering analgesics	4.87	0.34	0.07	0.775^b^	0.0076	1
	• A-3 conduct pain assessment after administering analgesics	4.83	0.39	0.08	0.790^b^	0.0052	0.5
	• A-4 conduct pain assessment upon patient admission or transfer to another department.	4.87	0.46	0.09	0.766^b^	0.0076	1
	• A-5 conduct pain assessment during each shift.	4.57	0.73	0.16	0.702^b^	0.0026	0.5
	• A-6 pain location	5.00	0	0	0.751^b^	0.0076	1
	• A-7 pain intensity	5.00	0	0	0.777^b^	0.0076	1
	• A-8 temporal characteristics of pain (e.g., onset and duration)	4.96	0.21	0.04	0.775^b^	0.0044	0.5
	• A-9 nature of pain	5.00	0	0	0.752^b^	0.0076	1
	• A-10 factors aggravating or alleviating pain	4.96	0.21	0.04	0.716^b^	0.0044	0.5
	• A-11 impact of pain on the patient's quality of life (e.g., sleep)	4.87	0.34	0.07	0.707^b^	0.0031	0.5
	• A-12 use validated and reliable pain assessment tools	5.00	0	0	0.705^b^	0.0196	2
	• A-13 select a self-report pain assessment tool for patients able to communicate	4.91	0.29	0.06	0.696^b^	0.0148	1.5
	• A-14 use objective pain assessment tools for patients unable to communicate	4.83	0.39	0.08	0.652^b^	0.0088	1
	• A-15 conduct pain assessment based on patient feedback, incorporating a physical examination when necessary	*4.87*	*0.34*	*0.07*	*Excluded*	*0.0114*	*/*
	• A-16 empathize with the patient's feelings	4.52	0.59	0.13	0.511^b^	0.0044	0.5
	• A-17 conduct individualized assessments based on the patient's clinical condition, age, communication level, and cognitive function	4.96	0.21	0.04	0.612^b^	0.0083	1
	• A-18 continuously monitor the patient's pain condition	4.87	0.34	0.07	0.596^b^	0.0059	0.5
	• A-19 reassess pain regularly using appropriate tools	4.83	0.39	0.08	0.640^b^	0.0035	0.5
	• A-20 document the pain assessment tools used and assessment results	4.83	0.39	0.08	0.627^b^	0.0035	0.5
Dimension B: assessment and management of movement-evoked pain and unexpected pain	• B-1 assess movement-evoked pain before and during functional exercises	4.96	0.21	0.04	0.411^d^	0.0107	1
	• B-2 assess movement-evoked pain when the patient reports pain interfering with functional activities	5.00	0	0	0.417^d^	0.0214	2
	• B-3 Use the Functional Activity Score (FAS) as the assessment tool for movement-evoked pain	4.96	0.21	0.04	0.626^d^	0.0107	1
	• B-4 for FAS grade a: guide the patient in proper functional exercises	4.91	0.29	0.06	0.696^d^	0.0107	1
	• B-5 for FAS grade B: administer analgesics as prescribed and guide functional exercises	4.91	0.29	0.06	0.690^d^	0.0107	1
	• B-6 for FAS grade C: report to the physician and adjust analgesic treatment per medical orders	4.7	1.06	0.23	0.670^d^	0.0054	0.5
	• B-7 when uncontrolled pain occurs	4.96	0.21	0.04	0.446^d^	0.0231	2
	• B-8 when sudden severe pain occurs	4.96	0.21	0.04	0.406^d^	0.0231	2
	• B-9 when progressively worsening pain occurs	5.00	0	0	0.405^d^	0.0462	4.5
	• B-10 monitor vital signs and observe potential complications (e.g., massive bleeding) and comorbidities.	4.96	0.21	0.04	0.427^d^	0.0239	2.5
	• B-11 conduct physical examination when necessary	*4.78*	*0.42*	*0.09*	*Excluded*	*0.0119*	*/*
	• B-12 report to the physician and follow medical instructions for management	4.96	0.21	0.04	0.292^d^	0.0239	2.5
Dimension C: pharmacological pain management	• C-1 administer analgesics as prescribed	4.91	0.29	0.06	0.659^a^	0.0184	2
	• C-2 ensure oral opioids are properly administered	4.78	0.52	0.11	0.642^a^	0.0066	0.5
	• C-3 use transdermal analgesic formulations correctly	4.83	0.39	0.08	0.648^a^	0.0109	1
	• C-4 evaluate analgesic efficacy at appropriate intervals based on the administration route	4.83	0.49	0.10	0.697^a^	0.0109	1
	• C-5 monitor respiratory depression and sedation levels when using opioids, while monitoring constipation, nausea, vomiting, and urinary retention; report any abnormalities to the physician	5.00	0	0	0.654^a^	0.0368	3.5
	• C-6 monitor gastrointestinal side effects and liver/kidney function when using NSAIDs; report any abnormalities	4.96	0.21	0.04	0.672^a^	0.0184	2
	• C-7 monitor for depression, ataxia, dizziness, drowsiness, and respiratory depression when using neuropathic pain medications (e.g., pregabalin)	4.96	0.21	0.04	0.619^a^	0.0184	2
	• C-8 identify the cause of analgesic adverse reactions and rule out other contributing factors	4.91	0.29	0.06	0.650^a^	0.0047	0.5
	• C-9 administer preventive medications as prescribed and evaluate efficacy	4.87	0.34	0.07	0.709^a^	0.0029	0.5
	• C-10 for vomiting: report to the physician, monitor electrolyte imbalances, and rule out bowel obstruction, gastric dilation, or increased intracranial pressure	4.91	0.29	0.06	0.711^a^	0.0047	0.5
	• C-11 for bloating and constipation: encourage early mobilization, provide abdominal massage, and implement prescribed treatments	4.87	0.34	0.07	0.770^a^	0.0029	0.5
	• C-12 for urinary retention: apply warm compresses; use enemas, Traditional Chinese Medicine (TCM) therapies, or catheterization as ordered	4.74	0.45	0.09	0.751^a^	0.0020	0.5
	• C-13 for excessive sedation or respiratory depression: immediately report to the physician, discontinue opioids/sedatives, and assist with emergency treatment	5.00	0	0	0.733^a^	0.0075	1
	• C-14 evaluate the effectiveness of adverse reaction management	4.91	0.29	0.06	0.729^a^	0.0047	0.5
Dimension D: patient-controlled analgesia (PCA) management	• D-1 secure tubing and ensure proper device function	4.96	0.21	0.04	0.762^a^	0.0342	3.5
	• D-2 instruct patients to press the PCA button when experiencing pain, and preemptively press 5–10 min before activity	4.91	0.42	0.08	0.753^a^	0.0171	2
	• D-3 conduct shift handovers and double-check the PCA pump's status, tubing condition, dosage, and ineffective attempts	4.91	0.29	0.06	0.718^a^	0.0171	2
	• D-4 evaluate vital signs, level of sedation (LOS), and analgesic effects every 4–8 h, monitoring for complications such as numbness or progressive muscle weakness	4.91	0.29	0.06	0.685^a^	0.0171	2
	• D-5 report inadequate analgesia to the physician or acute pain service (APS)	4.87	0.34	0.07	0.655^a^	0.0086	
	• D-6 identify and address common PCA issues (e.g., alarms, tubing blockage, empty medication reservoirs)	4.87	0.34	0.07	0.699^a^	0.0086	1
Dimension E: non-pharmacological pain management	• E-1 apply non-pharmacological measures independently for mild pain	4.52	0.95	0.21	0.731^a^	0.0086	1
	• E-2 combine non-pharmacological measures with analgesics for moderate to severe pain	4.83	0.39	0.08	0.741^a^	0.0259	2.5
	• E-3 provide individualized non-pharmacological pain interventions based on patient condition and preferences	4.83	0.39	0.08	0.717^a^	0.0052	0.5
	• E-4 assist the patient in adopting pain-preventive functional positions	4.74	0.54	0.11	0.746^a^	0.0034	0.5
	• E-5 utilize non-pharmacological interventions such as deep breathing, massage, distraction, cold/heat therapy, or relaxation training	4.65	0.57	0.12	0.757^a^	0.0025	0.5
	• E-6 incorporate Traditional Chinese Medicine methods (e.g., auricular acupressure) as appropriate	*4.35*	*0.83*	*0.19*	*Excluded*	*0.0011*	*/*
	• E-7 provide psychological support and sleep guidance when necessary	*4.57*	*0.79*	*0.17*	*Excluded*	*0.0017*	*/*
	• E-8 evaluate intervention effectiveness and adverse reactions at appropriate intervals	4.74	0.75	0.16	0.685^a^	0.0034	0.5
Dimension F: pain education	• F-1 educate patients on the importance and timing of reporting pain	4.96	0.21	0.04	0.764^a^	0.0152	1.5
	• F-2 teach patients to use appropriate pain self-assessment tools	4.91	0.29	0.06	0.768^a^	0.0097	1
	• F-3 explain the adverse effects of pain and the importance of pain assessment	4.83	0.49	0.10	0.760^a^	0.0061	0.5
	• F-4 educate patients on postoperative pain complications and the need for timely reporting	4.91	0.29	0.06	0.764^a^	0.0203	2
	• F-5 emphasize early mobilization and pain control during functional activities	4.96	0.21	0.04	0.761^a^	0.0406	4
	• F-6 explain medication usage and potential side effects	4.83	0.39	0.08	0.774^a^	0.0121	1
	• F-7 provide instruction on PCA device operation and related precautions	4.87	0.34	0.07	0.736^a^	0.0170	2
	• F-8 teach non-pharmacological techniques and related precautions	4.70	0.76	0.16	0.758^a^	0.0061	0.5
	• F-9 highlight the importance of patient and family involvement in pain management	*4.78*	*0.67*	*0.14*	*Excluded*	*0.0087*	*/*
	• F-10 provide instructions on medication use and precautions	4.83	0.49	0.10	0.743^a^	0.0344	3.5
	• F-11 explain the necessity and methods of non-pharmacological interventions	4.65	0.71	0.15	0.740^a^	0.0172	2
	• F-12 educate patients on when to seek prompt medical attention for inadequate pain relief, complications, or unexpected pain	4.83	0.39	0.08	0.726^a^	0.0344	3.5
Dimension G: professional development competencies	• G-1 use the SBAR (situation–background–assessment–recommendation) model for communication between healthcare providers	4.74	0.69	0.15	0.453^c^	0.0068	0.5
	• G-2 clarify roles of multidisciplinary pain management team members and seek assistance as needed	4.70	0.56	0.12	0.551^c^	0.0048	0.5
	• G-3 demonstrate basic triage skills	*4.61*	*0.58*	*0.13*	*Excluded*	*0.0024*	*/*
	• G-4 participate in multidisciplinary team (MDT) discussions on pain-related issues	4.65	0.49	0.10	0.773^c^	0.0035	0.5
	• G-5 apply evidence-based practices (e.g., literature review) in pain management nursing	4.70	0.47	0.10	0.829^c^	0.0307	3
	• G-6 explore and implement new methods/technologies in pain management nursing	4.61	0.50	0.11	0.852^c^	0.0154	1.5
	• G-7 understand the content of pain management quality improvement indicators and audit methods.	4.78	0.52	0.11	0.832^c^	0.0083	1
	• G-8 participate in data collection and analysis for quality improvement projects	4.83	0.49	0.10	0.846^c^	0.0131	1.5
	• G-9 propose recommendations to improve pain management practices	4.61	0.58	0.13	0.828^c^	0.0053	0.5
	• G-10 proactively analyze the causes of pain and determine its etiology from a holistic care perspective	4.83	0.39	0.08	0.772^c^	0.0038	0.5
	• G-11 proactively implement nursing interventions to alleviate patients' pain	4.91	0.29	0.06	0.695^c^	0.0060	0.5
	• G-12 provide constructive recommendations to physicians	4.74	0.45	0.09	0.642^c^	0.0024	0.5

SD, standard deviation; CV, coefficient of variation.

^a^, ^b^, ^c^, and ^d^ correspond to Factor 1, Factor 2, Factor 3, and Factor 4, respectively, in the exploratory factor analysis.

Italicized items indicate those deleted in Phase 2 (Measurement Validation and Application).

Fifteen nurses participated in the pilot study. The questionnaire's completion time ranged from 6 to 22 min (average: 9 min). The 14 items in the SNPMC were rephrased to enhance clarity and contextual relevance based on participant feedback.

### 3.2 Measurement validation and application results (phase 2)

#### 3.2.1 Characteristics of sampling hospitals and participants

Fifteen provinces, municipalities, and autonomous regions were selected: (a) eastern economic region: Guangdong, Shandong, Zhejiang, and Shanghai municipalities; (b) central economic region: Henan, Hubei, Jilin, Jiangxi, and Shaanxi provinces, the Inner Mongolia Autonomous Region, and Chongqing Municipality; and (c) western economic region: Gansu, Guizhou, and Yunnan provinces and the Xinjiang Uyghur Autonomous Region.

This study included 48 hospitals: 19 from the eastern economic region (39.6%), 18 from the central economic region (37.5 %), and 11 from the western economic region (22.9%).

Of the 1,974 surgical nurses surveyed, 1,885 provided valid responses. The number of participants per hospital ranged from 3 to 195 (average: 39/hospital). The participants were distributed throughout the eastern (39.4%), central (37.5%), and western (23.1%) economic regions.

Most participants were from tertiary hospitals (86.4%) and held a bachelor's degree (84.8%). Nearly half of the respondents were aged 30–39 years (49.8%), and 44.1% had less than 10 years of professional experience. A detailed breakdown of the participants' demographic and professional characteristics is provided in Table 4. The distribution across departments was as follows: orthopedics (46.6%), general surgery (26.9%), obstetrics and gynecology (7.3%), urology (5.6%), cardiothoracic surgery (3.4%), and neurosurgery (3.3%).

**Table 4:** Nurses' pain management competence status and associated factors (*N* = 1,885).

**Variable**		***n* (%)**	**SNPMC score**	***t/F***	***p*-Value**	**Multi compare**
Economic region	Eastern^a^	742 (39.4)	82.8 ± 27.8	17.122	<0.001	a, b > c
	Central^b^	707 (37.5)	84.6 ± 26.2			
	Western^c^	436 (23.1)	74.8 ± 32.1			
Hospital tier	Tertiary hospital	1,628 (86.4)	82.0 ± 28.4	1.403	0.161	
	Secondary hospital	257 (13.6)	79.3 ± 29.3			
Sex	Male	32 (1.7)	82.4 ± 27.7	0.153	0.878	
	Female	1,853 (98.3)	81.6 ± 28.6			
Age (years)	<30^a^	600 (31.8)	75.6 ± 32.5	19.965	<0.001	a < b, c
	30–39^b^	939 (49.8)	84.1 ± 26.5			
	≥40^c^	346 (18.4)	85.3 ± 24.5			
Seniority (years)	<10^a^	831 (44.1)	77.5 ± 31.6	16.057	<0.001	a < b, c
	10–15^b^	657 (34.9)	84.2 ± 26.4			
	>15^c^	397 (21.1)	85.9 ± 23.7			
Education	Associate degree^a^	219 (11.6)	76.6 ± 33.3	6.916	0.001	a, c < b
	Bachelor's degree^b^	1,598 (84.8)	82.6 ± 27.6			
	Postgraduate degree^c^	68 (3.6)	74.0 ± 31.8			
Professional title	Entry-level^a^	836 (44.4)	77.7 ± 31.8	14.688	<0.001	a < b, c
	Intermediate-level^b^	868 (46)	84.6 ± 25.7			
	Senior-level^c^	181 (9.6)	85.6 ± 22.6			
Job position	Bedside nurse	1,640 (87)	81.4 ± 29.0	1.127	0.324	
	Nurse educator or team leader	94 (5)	85.9 ± 24.3			
	Nurse manager	151 (8)	81.5 ± 25.3			
Department	Obstetrics and gynecology^a^	138 (7.3)	75.3 ± 33.1	5.052	<0.001	b, c, e, f > g
	Colorectal surgery^b^	50 (2.7)	89.1 ± 19.4			
	Orthopedics^c^	878 (46.6)	84.0 ± 26.8			
	Head and neck-breast surgery^d^	79 (4.2)	79.6 ± 32.2			
	Urology^e^	105 (5.6)	82.6 ± 24.7			
	General Surgery^f^	508 (26.9)	80.8 ± 28.6			
	Neurosurgery^g^	62 (3.3)	66.8 ± 39.6			
	Cardiothoracic Surgery^h^	65 (3.4)	78.6 ± 29.2			
Is annual pain management training provided to nurses at the hospital?	No	455 (24.1)	77.4 ± 31.2	3.650	<0.001	
	Yes	1,430 (75.9)	83.0 ± 27.5			
Have you participated in any pain management continuing education or academic conferences in the past 3 years?	No	1,124 (59.6)	78.7 ± 30.5	5.372	<0.001	
	Yes	761 (40.4)	85.9 ± 24.8			

The superscript letters (a-h) denote the subgroups used in post-hoc multiple comparisons.

#### 3.2.2 Psychometric analysis of the SNPMC

##### 3.2.2.1 Content validity

Five experts who participated in the Delphi consultation assessed the content validity. They had a mean age of 47.6 ± 3.3 years and a mean of 21.2 ± 7.6 years of professional experience. Three were in nursing management, and two specialized in pain-management nursing. Additionally, two held PhDs, two had master's degrees, and one had a bachelor's degree. The I-CVI ranged from 0.96 to 1.00, and the S-CVI was 0.98, indicating excellent content validity.

##### 3.2.2.2 Construct validity

The KMO coefficient (0.991) and Bartlett's test of sphericity (*p* < 0.001) confirmed the appropriateness of the sample. Factor extraction and rotation were performed using principal axis factoring and the varimax method. Table 3 displays the detailed factor structure and rotated component matrix resulting from this analysis. Based on these results, items with factor loadings < 0.40 were excluded, resulting in the removal of six items (A-15, B-11, E-6, E-7, F-9, and G-3). Item B-12, despite not meeting the loading threshold, was retained owing to its clinical importance and relevance to practice.

This analysis served to refine the scale structure rather than confirm a theoretical model. The EFA extracted four factors with eigenvalues ranging from 5.552 to 25.730, accounting for 7.12%−32.99% of the total variance after rotation. The total variance accounted for 75.29%. Factor 1 comprised items from four dimensions: pharmacological pain management (dimension C), PCA management (dimension D), nonpharmacological pain management (dimension E), and pain education (dimension F). Factor 2 comprised items from routine pain assessment (dimension A); Factor 3 comprised items from professional development competencies (dimension G); and Factor 4 comprised items from the assessment and management of movement-evoked and unexpected pain (dimension B).

##### 3.2.2.3 Reliability

The overall Cronbach's α and McDonald's ω were 0.991 and 0.992, respectively. All seven dimensions demonstrated excellent internal consistency. Cronbach's α ranged from 0.946 to 0.983, and McDonald's ω ranged from 0.947 to 0.983. The two reliability estimates were nearly identical across dimensions, indicating that conclusions about reliability were consistent regardless of whether α or ω was used. By dimension, reliability estimates were as follows: A (α = 0.981, ω = 0.981), B (α = 0.966, ω = 0.967), C (α = 0.975, ω = 0.976), D (α = 0.946, ω = 0.947), E (α = 0.961, ω = 0.962), F (α = 0.983, ω = 0.983), and G (α = 0.969, ω = 0.971).

Test–retest reliability was assessed using data from 28 female surgical nurses who completed the scale twice within a 2-week interval. Their mean age was 33.2 ± 6.4 years, and their mean work experience was 10.7 ± 7.0 years. The Pearson correlation coefficient between the assessments was 0.493 (*p* = 0.008), indicating acceptable test-retest reliability. The intraclass correlation coefficient [ICC_(3, 1)_; two-way mixed-effects model, single measures, consistency] was 0.659 (95% CI = 0.262–0.842; *p* = 0.003), indicating moderate test–retest reliability. Using SD = 41.5, the standard error of measurement (SEM) was 29.6, the smallest detectable change for an individual was 82.1, and the group-level SDC was 15.5.

#### 3.2.3 Final tool structure and scoring method

The finalized SNPMC comprised 78 items, each rated on a five-point Likert scale ranging from 1 (“not competent at all”) to 5 (“completely competent”). The item scores were weighted using the coefficients derived from the AHP (Table 3). This weighting resulted in individual item scores from 0.5 to 4.5. The total score ranged from 0 to 100, with higher scores indicating greater competency in pain management among surgical nurses. The floor effect was negligible, with only 3.66% (69/1,885) of participants scoring the minimum. However, a ceiling effect was observed, with 26.10% (492/1,885) of participants achieving the maximum score of 100. To facilitate the clinical interpretation of SNPMC scores, normative percentile bands were calculated based on the distribution of the study sample. The 25th percentile (*P*_25_), median (*P*_50_), and 75th percentile (*P*_75_) scores were 80.5, 95.5, and 100.0, respectively. Given the high overall proficiency and the observed ceiling effect, we propose the following interpretive guidelines: Basic Competence (Needs Improvement): Total Score < 80.5; Moderate Competence: Total Score 80.5–95.5; Advanced Competence: Total Score >95.5.

#### 3.2.4 Current status of nurses' pain-management competency and its associated factors

Survey data from 1,885 surgical nurses indicated a mean SNPMC score of 81.6 ± 28.5. Table 4 summarizes the exploratory subgroup comparisons, highlighting significant disparities associated with regional and professional characteristics. Significant regional differences were observed (*F* = 17.122, *p* < 0.001), with nurses in the eastern (82.8 ± 27.8) and central (84.6 ± 26.2) regions scoring higher than those in the western region (74.8 ± 32.1). Other factors significantly associated with higher competency scores included greater seniority (*p* < 0.001), higher professional titles (*p* < 0.001), and holding a bachelor's degree (*p* = 0.001).

Additionally, engagement in annual hospital training (*p* < 0.001) or continuing education (*p* < 0.001) was linked to better pain-management competency performance. No significant differences were found for hospital tier, sex, or job position (*p* > 0.05).

## 4 Discussion

### 4.1 Scientific development and psychometric properties of the SNPMC

Nurses play a central role in pain management, and several tools, such as the NCPMCS, have been developed to evaluate their competency in specific contexts such as cancer pain. However, postoperative pain differs from cancer pain in its onset, duration, and management strategies, highlighting the necessity for a targeted assessment tool. To our knowledge, the SNPMC is among the few instruments specifically designed to assess postoperative pain management competency in surgical nursing and extends existing tools by incorporating surgical workflow-specific requirements. This tool can support competency-based assessment and targeted training within surgical systems.

This study employed a rigorous and systematic approach to develop the SNPMC, ensuring scientific rigor and clinical relevance. The development process included a comprehensive literature review, semi-structured interviews, expert discussions, Delphi expert consultations, and pre-testing. Qualitative interviews enriched the initial item pool and grounded the instrument in real-world clinical practice. The Delphi process involved 23 experts from various nursing fields across 13 provinces and cities. Through two rounds of consultation, the experts refined the instrument's dimensions and items. All methodological requirements for the Delphi consultation were met, achieving a high degree of consensus (27).

The final instrument demonstrated strong psychometric properties, with an overall Cronbach's α coefficient of 0.991, indicating high internal consistency, and a scale-level content validity index (S-CVI) of 0.98, confirming strong content validity. All 78 items, excluding one (B-12), which was retained owing to its importance for clinical experience, had factor loadings above 0.4 (28), and the cumulative explained variance reached 75.29%, surpassing the accepted 50% threshold (28). These results suggest that the SNPMC is a valid and reliable instrument for assessing surgical nurses' postoperative pain-management competency. Given that the dimensional structure was derived using EFA, the identified dimensions should be interpreted as empirically observed patterns rather than a finalized theoretical model.

Meanwhile, a ceiling effect (26.10%) was noted, suggesting that the SNPMC items primarily capture essential competency standards well-mastered by a substantial proportion of experienced nurses in our sample. Although this confirms the tool's utility for verifying baseline competence and safety, it may have reduced sensitivity in distinguishing between high and very high levels of proficiency. Future versions could consider incorporating more advanced or complex case-based items to improve discrimination at the upper end. Furthermore, the instrument's high reliability combined with the ceiling effect suggests a degree of item redundancy. Although the comprehensive nature of the 78-item scale is valuable for detailed educational assessment, it may be burdensome in busy clinical settings. Future research should therefore prioritize the development of a statistically rigorous short-form version using Item Response Theory (IRT) to retain maximal information with fewer items, thereby improving feasibility for routine screening.

To support score interpretation, percentile-referenced normative bands derived from the sample distribution provide tiered benchmarks for identifying relatively lower competency, but they should be treated as context-specific guidelines requiring external validation.

### 4.2 Multidimensionality of the SNPMC: reflecting practice requirements and surgical pain-management characteristics

The SNPMC comprises 78 items across seven dimensions: (a) routine pain assessment, (b) assessment and management of movement-evoked pain and unexpected pain, (c) pharmacological pain management, (d) PCA management, (e) non-pharmacological pain management, (f) pain education, and (g) professional development competencies. These dimensions collectively reflect the core practices required for postoperative pain management, while highlighting the specific characteristics and challenges of surgical care.

The dimensions assessment and management of movement-evoked pain and unexpected pain, and pain education carried the highest weights (0.1498 each), whereas non-pharmacological pain management had the lowest (0.0517). Hence, the SNPMC captures general pain-management practices while emphasizing areas of particular relevance to the surgical context.

Movement-evoked pain and unexpected pain are two common yet distinct forms of postoperative pain that require specialized nursing care (29, 30). Movement-evoked pain, typically more severe and difficult to control than pain at rest, requires accurate assessment using appropriate tools and timely interventions to support functional recovery and reduce complications. In contrast, unexpected pain refers to sudden, unanticipated pain episodes that may signal underlying postoperative complications, such as infection, hematoma, or displacement of internal fixation devices. Effective management requires nurses to maintain a high level of clinical vigilance, rapidly identify potential causes, and take appropriate actions. These tasks reflect the critical judgment and responsiveness required of surgical nurses in complex postoperative settings.

The importance of pain education is likely related to the growing emphasis on patient-centered care. Inconsistent pain education across healthcare providers can lead to variations in care quality (31). As frontline educators, nurses are essential in educating patients and families to improve pain recognition, treatment adherence, and recovery outcomes (32).

PCA management (factor weight = 0.1027) also holds significant importance, particularly as PCA is frequently utilized in postoperative settings, making its application a critical responsibility of surgical nurses. Recognizing this, the SNPMC treats PCA care as an independent competency domain, reflecting its technical specificity and importance in surgical pain-management quality.

In summary, the SNPMC provides a structured and multidimensional framework that aligns with evidence-based pain nursing requirements and the nuanced demands of surgical settings. It is a valuable tool for identifying strengths and gaps in nurses' competencies and guiding targeted education and training programs.

### 4.3 Innovation of the SNPMC and comparison with existing instruments

The SNPMC demonstrates clear innovation in its focus, structure, and clinical applicability. Although many tools assess nursing competencies in pain management, most target different populations or emphasize various pain contexts without a comprehensive surgical nursing perspective. For example, Hu et al. (13) developed the NCPMCS, highlighting four key attributes of cancer pain-management competency: context of pain management, pain assessment and measurement, management of pain, and multidimensional nature of pain. Despite offering a macro-level understanding of nursing competencies in cancer pain, it includes chronic and end-of-life pain, which are not directly applicable to the acute, dynamic postoperative pain in surgical settings. Thus, its clinical relevance for surgical nurses is limited. Meanwhile, Herr et al. (15) proposed 21 pain-management competencies for nursing students to guide pre-licensing education, without direct relevance for registered nurses who operate in complex, high-demand clinical environments such as surgical wards. Additionally, the Knowledge and Attitudes Survey Regarding Pain, developed by Ferrell and McCaffery (33), is widely used to assess nurses' pain-related knowledge and attitudes based on clinical standards. However, it does not evaluate competency in practical skills, confidence, or decision-making, essential components of clinical competency. Still further, Li et al. (20) designed a tool to assess evidence-based nursing practices for pain management. However, it solely focuses on assessment, excluding the full spectrum of pain-management tasks like intervention, education, and professional development. The SNPMC further supports clinical utility by providing interpretable total and domain-level scores that can inform training prioritization.

### 4.4 Analysis of national survey results and associated factors of surgical nurses' pain-management competency

This large-scale, nationally representative survey of 1,885 surgical nurses from three economic regions in China revealed moderate to high pain-management competency levels, with an overall mean score of 81.6 ± 28.5. Eight factors were identified as significantly associated with this competency: economic region, annual pain-management training, participation in advanced pain-management studies or academic conferences, age, years of work experience, educational level, professional title, and department.

Regional disparities were evident, with nurses in the central economic region demonstrating the highest average competency scores while those in the western region scored the lowest. These differences may be related to variations in access to continuing education opportunities, institutional expectations, and resource availability. Nurses working in economically developed regions may benefit from more frequent training and a stronger emphasis on professional development, enhancing their pain-management capabilities. Indeed, nurses who had received annual pain-management training or participated in advanced studies or academic conferences tended to exhibit higher pain-management competency scores. These findings align with those of previous studies by Vu et al. (34) and Bernardi et al. (35), which identified insufficient pain education as a significant barrier to effective pain management in clinical settings. Hence, targeted training programs can significantly improve nurses' knowledge and practical applications of postoperative pain management.

Demographic and professional variables, such as age, years of clinical experience, education level, and department, were also significantly correlated with competency scores. This aligns with the findings of Admassie et al. (36), which indicated that older age, higher education, more years of work experience, and prior pain-management training positively correlated with nurses' knowledge of and attitudes toward pain management. Because our study was a cross-sectional survey, the observed associations should not be interpreted as causal relationships.

Based on these findings, it is recommended that strategic interventions be implemented at the macro- and meso-levels to enhance surgical nurses' pain-management competencies. At the macro-level, national healthcare policies should prioritize resource allocation to underdeveloped regions, especially the less developed western provinces of China. Cross-regional support mechanisms, such as partnerships between hospitals in economically advanced eastern regions and those in the West, could help bridge educational and training gaps, thereby improving continuous education access for nurses in underserved areas. At the meso- (institutional) level, hospitals should emphasize cultivating pain-management competencies among nursing staff. Support mechanisms, including funding for training, opportunities for external studies, and professional recognition, should be established to motivate nurses interested in pain care. These efforts can cultivate a culture of continuous learning and competency enhancement, ultimately leading to improved pain management in surgical care settings. Future implementation research may examine associations between SNPMC domain scores and relevant process indicators (e.g., reassessment adherence and PCA safety-check compliance) and functional recovery-related metrics to inform quality improvement.

### 4.5 Strengths and limitations

The SNPMC offers a detailed and context-specific framework for assessing postoperative pain management in surgical settings. Its multidimensional structure captures a wide range of competencies required in practice, addressing evolving expectations of surgical nurses. Integrating domains such as movement-evoked pain management, PCA care, and pain education, the SNPMC fills gaps left by earlier instruments, providing a more holistic and practice-oriented approach to competency assessment.

This study had some limitations.

First, we acknowledge that the content validity assessment was conducted with a small panel of only five experts, some of whom overlapped with contributors to our Delphi process. This panel composition risks inflating the Content Validity Index (CVI) estimates, whereas best practice recommends a larger, independent panel and metrics such as Lawshe's CVR. Although the instrument's items were established through a robust foundational phase, including two Delphi rounds with 23 experts, we consider this CVI evaluation step a clear limitation. We therefore recommend that future studies employ a larger and fully independent expert panel to corroborate the content validity of the SNPMC. Additionally, conducting a sensitivity analysis to compare the performance of weighted vs. unweighted scoring would provide valuable insight into the clinical utility of the AHP-derived weights.

Second, a key limitation is the discrepancy between the four-factor structure that emerged from our EFA and our theoretically derived seven-dimension framework. This may be partly due to methodological choices, such as using an orthogonal (varimax) rotation—which assumes uncorrelated dimensions—and treating ordinal Likert data as continuous. Although we retained the seven-dimension model for its strong clinical and educational utility, this conflict underscores the need for future research to employ Confirmatory Factor Analysis (CFA) to formally test the proposed structure, potentially alongside more advanced EFA techniques (e.g., oblique rotation with polychoric correlations).

Third, the instrument's extremely high reliability suggests item redundancy, a limitation for the practical use of this comprehensive 78-item scale. Consequently, future research must prioritize developing a validated short form. Applying item reduction techniques, such as Item Response Theory (IRT), is essential to create a more feasible tool for clinical settings without compromising reliability.

Fourth, test-retest analysis was conducted with a single-center sample, which limits the generalizability of the stability findings. As our primary focus was on the initial content development of the instrument, future validation studies must employ a larger, multi-center sample to more robustly establish the SNPMC's stability across diverse clinical settings. Meanwhile, the percentile-based interpretive bands reported here are sample-derived and require replication before being used for cross-institutional benchmarking.

Fifth, our use of convenience sampling restricts the generalizability of our findings. This non-probability method may have introduced selection biases, such as over-representing nurses from larger or more research-active hospitals. Therefore, our results should be interpreted with caution as they may not be fully representative. Corrective techniques like post-stratification weighting were considered but were not feasible due to a lack of available population benchmarks.

Lastly, this foundational study did not include advanced validation analyses. We did not assess convergent, discriminant, or criterion validity, nor did we test for measurement invariance across groups. Therefore, the instrument's relationship to other constructs, its predictive utility, and the fairness of group comparisons remain to be established. These advanced psychometric evaluations are a critical priority for future research to fully support the SNPMC's use in practice.

### 4.6 Recommendations for future research

Although this study relied on Exploratory Factor Analysis (EFA), we recognize that competency constructs often exhibit complex structures that strict independent-clusters models may not fully capture. Future research using independent samples should employ modern confirmation strategies, such as semi-confirmatory factor analysis or bifactor modeling, to better address cross-loadings and disentangle general competency variance from domain-specific variance (e.g., distinguishing broad pain management ability from specific technical skills like PCA management). Furthermore, future validation efforts should move beyond self-report measures by incorporating objective criterion indicators, such as patient outcomes derived from automated or multimodal pain assessment technologies, to robustly establish the instrument's real-world predictive validity. Cross-cultural adaptation and invariance testing are also needed before applying the SNPMC in non-Chinese contexts.

## 5 Conclusions

This study developed and validated the SNPMC, a reliable instrument for assessing pain-management competency among surgical nurses, a population previously overlooked by existing tools. As the first tool specifically tailored to this group, the SNPMC fills an important gap created by existing assessment tools. It provides a comprehensive framework encompassing seven dimensions ranging from routine pain assessment to professional development.

The SNPMC provides interpretable total and domain-level competency scores to support targeted training and internal quality improvement. In the national survey, surgical nurses showed moderate-to-high pain-management competency levels, which were associated with clinical experience, education, and prior training. External validation, including cross-institutional and cross-cultural evaluation, is needed before broader benchmarking or application beyond the current context.

## Data Availability

The datasets presented in this article are not readily available because the datasets generated and/or analysed during the current study are not publicly available but are available from the corresponding author on reasonable request. Requests to access the datasets should be directed to Yunxia Li, 3194083@zju.edu.cn.
